# The County Council and Public Health

**Published:** 1907-03-30

**Authors:** 


### THE COUNTY COUNCIL AND PUBLIC HEALTH.

The County Council is the supreme authority in London, and, like all beneficent rulers, it takes a deep interest in the health of its subjects. Since it became the manager of the elementary schools, the Council has been forced to take special cognisance of those epidemic diseases which are chiefly spread through school children. Very little attention is paid by the ordinary Press or the public to a two-line notice that a certain school has been closed on account of an outbreak of measles or scarlet fever, and only those who have to do with the matter realise the work and responsibility involved in dealing with the epidemics thus briefly noted. The report of the medical officer of health for the County of London is, to those who will take the trouble to read it, a most interesting document, showing how much watch is kept over the health of Londoners, and how, unfortunately, in spite of all care, diseases manage to spread. By analysing the frequency and methods of epidemics one can sometimes arrive at the causes, and thus gain from the experience of the past, however fatal, some guidance for wisdom in the future.

The careful statistics which are now being gathered show that in many diseases there is a regular periodicity. Thus measles springs into fatal prominence in alternate years. The intervals between successive outbreaks is about eighteen months, and Sir Shirley Murphy thinks that these successive prevalences are associated with the accumulation of susceptible children. The infection in measles largely comes from the school, but the victims for the most part are not school children, but their younger brothers and sisters under two years of age. While the mortality from measles at other ages has been decreasing for the last fifty years, the mortality under the age of two years has increased—within the last quinquennium, indeed, has increased largely. Between 1851 and 1860 the measles mortality under one year was 162 per thousand deaths, and that under two years of age 366 per thousand. In the quinquennium ending 1905 the mortality at these ages was respectively 226 and 423 per thousand. At higher ages the mortality has decreased. Sir Shirley Murphy believes the shifting of measles mortality to the earliest years of life to be due to the increasing aggregation of population in London and to the increased opportunity of infection the result of the collection of children in large public schools. It is satisfactory to know that the order of the County Council applying the provisions of the Public Health Act to measles has had some use in impressing upon parents the importance of taking precautions to prevent infectious children associating with others. School teachers also have grasped the importance of informing medical officers of health as to children who are absent from school on account of measles, while the sanitary authorities, by the distribution of leaflets explaining what should be done in the case both of children who are attacked and of those who are exposed to the disease, have done much to limit the mortality of measles. The measures employed to check the effects of this disease are even now of considerable effect; but the importance attached to infection, and the measures taken to prevent it, will doubtless impress the imagination of children now at school, and will develop in them a sense of the duty of preventing the spread of infection.

At present the County Council insists that children suffering from measles must be excluded from school for at least a month, and that children who come from infected homes must also be excluded if they have not themselves had measles, but not otherwise. This seems to imply that children who have themselves suffered cannot carry infection. The provision is reasonable and considerate. If the inference that persons who have suffered from the disease cannot be bearers of infection is not justified, no doubt future statistics will prove the point. It must be admitted that in the matter of the death-rate from measles London does not come out as well as one could desire. Comparing it with the other European capitals, and with New York, for the last ten years, one finds that our death-rate from measles is higher than that of any other city except St. Petersburg; and there is but scant consolation in the knowledge that up to 1904 it was exceeded by that of Manchester, Hull, and Salford, while in 1905 Birmingham, Sheffield, Bristol, and Nottingham also had a worse record.

Scarlet fever is another disease which is closely associated with elementary schools. But it appears that holidays are more responsible for its prevalence than school days. In 1905 special notice was taken of this point, and it was found that the four weeks of holiday, and the four immediately following, showed a much higher average of cases at all ages than the weeks preceding. Scarlet fever epidemics, which largely swell the statistics of the metropolis, are often entirely local in their incidence, for there is no disease that seems to be more easily conveyed than scarlet fever, and if the infecting case be mild it is often unrecognised until others have suffered from coming in contact with it. In the poorer districts it seems often to be quite impossible to discover the source of infection. In Finsbury, Dr. Newman was able to discover the source of 265 out of 456 cases brought to his notice; but in Battersea Dr. M'Cleary could trace only 144 out of 801, and in Bermondsey Dr. Brown could explain only 120 cases out of 768. This difficulty of finding whence the infection came must form a serious difficulty in checking an outbreak. In Wandsworth, Dr. Caldwell Smith was fortunate in tracing twenty cases to one source—an infected milk supply. The liability of milk to convey infection is a problem by itself, which comes under the responsibilities of the County Council, but with which we cannot deal at this moment. Scarlet fever was rather more fatal in its effects during 1905 than in the preceding year, the deaths registered as due to it being 549, as against 365 in 1904. The rate for 1905 was the same as that for 1902—0.12 per thousand persons living, while the intervening years had the lowest death-rate recorded since the notification of infectious diseases was made compulsory in 1889. Notification does not, on the surface, seem to have had much influence in lessening the prevalence of the disease, for the case-rate per thousand living was 4.8 between 1891 and 1900, and 4.2 in 1905; but there has been a perceptible decrease in the proportion of deaths, which now stands at 2.8 per cent. of cases as against 3.9 at the beginning of the notification period; and it must also be remembered that there has been a growing carefulness among practitioners as to observing and isolating suspicious cases. The real difficulty in dealing with scarlet fever lies with uneducated parents—who are not by any means confined to any one class—those who do not call in a doctor to a case, especially if it be mild, until the patient has had time to injure those around. It is, however, satisfactory to see that a steadily increasing proportion of the cases notified are being treated in the hospitals belonging to the Metropolitan Asylums Board. In 1905 the proportion was over 85 per cent., which is about double what it was when notification first began. This shows that people are appreciating more and more the advantages of hospital treatment of infectious diseases—advantages which may be reckoned as equally divided between the patient who gets the treatment, and his family who escape the risk of infection.

